# Prevention and control of urinary tract stones using a smartphone-based self-care application: design and evaluation

**DOI:** 10.1186/s12911-021-01661-0

**Published:** 2021-11-01

**Authors:** Leila Shahmoradi, Amin Azizpour, Mahmud Bejani, Pejman Shadpour, Sorayya Rezayi

**Affiliations:** 1 Health Information Management and Medical Informatics Department, School of Allied Medical Sciences, Tehran University of Medical Sciences, Tehran, Iran; 2 Health Information Management, School of Management and Medical Informatics, Tabriz University of Medical Sciences, Tabriz, Iran; 3 Hasheminejad Kidney Center (HKC), Hospital Management Research Center (HMRC), Iran University of Medical Sciences, Tehran, Iran

**Keywords:** Applications, Mobile health, Self-care, Urinary tract stones, Evaluation

## Abstract

**Background:**

Self-care and participation of patients in improving health and increasing awareness about the risk factors that affect the development of disease in patients with urinary tract stones are influential factors in controlling and improving the quality of life in these patients. In this regard, the availability and capability of smartphones increase patients’ self-care ability. The present study aimed to develop and evaluate a self-care application based on smartphones for patients with urinary tract stones.

**Methods:**

The present study is a developmental and applied study that was conducted in three phases. First, the information needs and functionalities of the self-care application were determined by surveying 101 patients, 32 urologists and nephrologists, 11 nurses, and six other specialists. In the second phase, the initial sample of the smartphone-based application was created, and in the third phase, the designed application was evaluated by 15 experts using the standard Post-Study System Usability Questionnaire (PSSUQ 18.3) and Nielsen’s Attributes of Usability (NAU) questionnaire. Results of the questionnaires were entered into SPSS-23 software for analysis using descriptive statistics.

**Results:**

In the first phase, 21 information elements and nine critical functionalities for the self-care application were identified, and then this application was designed by Java programming language. The evaluation of experts showed that two aspects of the quality of system user interface from the user's point of view and the overall performance of the application together obtained the highest score (6.43 from 7), which was equal to 91.85%. Then according to the experts, aspects of the degree of convenience and user-friendliness of the application received the highest score (6.10 from 7), which was equal to 87.14%, and also all aspects of the application were evaluated at an acceptable level. In general, results of the evaluation of application's usability by experts showed that the usability of the application for patients with urinary tract stones was at an acceptable level.

**Conclusion:**

According to the results obtained from evaluating the smartphone-based application for patients with urinary tract stones, this self-care application can be used to prevent and control urinary tract stones and facilitate self-care and active patient participation in care.

## Background

Applying the right methods of health self-management is important in today’s world, when societies are faced with challenges and lack of resources. Meanwhile, among chronic diseases, the probability of developing kidney stone during one’s lifetime is estimated at 1 to 15% that varies according to age, sex, race and geographical area. In the United States, the prevalence of kidney stone disease is estimated at 10 to 15% [1]. In a recent National Health and Nutrition Examination Survey (NHANES), the overall prevalence of kidney stones was reported to be 10.6 in men and 7.1 in women. Accordingly, 1 in 11 people in the United States has a history of kidney stones, compared with 1 in 20 people in previous estimates [2, 3]. Other researchers have also observed this increase in the prevalence of kidney stones [4, 5]. Therefore, due to the significant increase in the prevalence of this chronic disease, the development of digital self-care interventions is needed, because the use of these interventions leads to an increase in people's health knowledge [6]. Also, beyond the boundaries of clinic and physician's office, self-care technologies enable patients to access the information they need at any time and place at the lowest possible cost. Self-care technologies are designed for different purposes (including education, support networks, self-management activities, etc.), and the necessary support can be adjusted according to the specific needs of patients. By changing the treatment and care needs of patients, their health behavior can be improved [7, 8].

The positive outcomes of digital self-care interventions include increased coverage, access, equity, and quality of services, reduced disparities and costs, and more efficient use of healthcare supplies and services. According to the World Health Organization (WHO) Guideline on Self-Care Interventions for Health and Well-Being, digital self-care interventions can help increase patients' active participation in their own health, including patient engagement in a supportive, safe, and patient-centered care environment [9]. Patient-centered mobile health technology interventions have the capacity and potential to improve self-care in patients with chronic disease [10]. These digital self-care applications allow health care professionals to extend their services by recommending to patients so that, most care-management processes are carried out by them by using these applications. Health care workers are likely to perceive mobile health technologies to be more efficient because they are portable and flexible [7]. Self-care mobile applications can provide self-management (i.e., self-medication, self-treatment, self-examination), self-testing (i.e., self-screening, self-diagnosis, self-monitoring), and self-awareness (i.e., self-help, self-education, self-regulation, self-efficacy) for patients [10].

Tapiero et al. [11] reviewed the application of smartphone technology in a review article and showed that, advances made in mobile health-related technologies and the increase in popularity of smartphones have provided a health promotion opportunity for patients and service providers. They also referred to the effectiveness of smartphone application in comparison to the performance of conventional methods. Consequently, the use of smartphone technology in urology education and care is growing and improving [12, 13]. However, Small AC et al., in a study of mobile-based emergency decision support system and management of patients with kidney stones, referred to the mobile-based application as a great tool for patients, health care providers and researchers [12, 14]. These new technology-based tools, which are relatively inexpensive, provide new opportunities to increase knowledge, education, and learning [15–19].

It is clear that applications based on mobile technologies have the necessary potential to improve disease management and health of patients [20]. Extensive studies have carried out worldwide to develop mobile-based health care tools and applications, with the aim of minimizing hospital interventions, reducing time and costs, and improving the quality of life of patients through chronic disease self-care applications [21]. Due to the increase in smartphone-based medical applications in recent years [22, 23], the extent to which these applications are used and downloaded from markets and application stores is a subject of interest for evaluation [24]. Also, the usefulness of these mobile-based health applications for disease management, self-monitoring, drug control, and clinical education purposes is important for patients, physicians, and health care providers, which increases the need for evaluation of these applications [25].

Due to the nature of care in patients with urinary tract stones and the lack of proper self-care package for these patients, and also taking into account the development of information technology in medicine, this study aimed to design and evaluate a smartphone-based application that provides informed and up-to-date self-care education to improve the information needs of patients with urinary tract stones and meet the unique needs of each patient such as type of stone and the specific conditions of each patient.

## Materials and methods

### Study overview

This developmental and applied study was conducted in Shahid Hasheminejad Hospital in Tehran in 2020 to design and evaluate a smartphone-based self-care application for patient with urinary tract stones. This study was performed in three main phases. In the first phase, information needs for design were determined based on studies and surveys of patients and experts. Consequently, in the second phase, based on the results of surveys, a self-care application was designed. Finally, in the third phase, this self-care application was evaluated by experts. Three main phases described in detail bellow:

### Identify information elements and functionalities of the application

For this purpose, relevant information sources for patients with urinary tract stones were collected and reviewed in terms of educational information and content and also the features and capabilities that these applications can have to help increase the awareness of these patients and healthy people in the community about this disease and how to prevent it. At the initial stages of this study, by reviewing scientific sources, including urology-related reference books and educational pamphlets, a researcher-made initial draft of questionnaire was created with 41 questions. To formulate the extracted information elements, the draft consisted of the following three sections: 1—information elements, 2—education and awareness on the urinary tract stones, and 3—functionalities of the application; this draft was distributed among ten faculty members of Shahid Hasheminejad Hospital as well as 12 urology residents of Shahid Hasheminejad Hospital; they were chosen as samples by convenience sampling method. Experts reviewed the initial draft to focus on the options and determine the necessity or non-necessity of information elements. At this stage of the research, the initial questionnaire was adjusted based on the content validity index (CVI) to assess the validity of the content and was given to experts. After making their desired corrections to simplify the specialized terminology used in the questions and the principled classification of the components, the content validity was assessed quantitatively through the content validity ratio (CVR). However, the questionnaire' reliability was obtained by Cronbach's alpha test of 85%.

After completing the above steps and summarizing the views of the research team, the items of the questionnaire were corrected and clarified. Finally, 21 information elements and nine functionalities that were more significant were selected to remain. The last version of the questionnaire was distributed among experts and patients. The scoring scale in this questionnaire was considered from one to four (Likert scale with four points), of which "score one" is for the least important element (i.e., unnecessary or impractical) and "score four" is for the most important element (i.e., very necessary or very practical). The participants in this survey were chosen using convenience sampling; during the study, 101 patients with various urinary tract stones were referred to Shahid Hasheminejad Hospital, 32 urologists and nephrologists working in Shahid Hasheminejad Center, and 11 nurses and six persons from other specialties were elected (totally 150 participants). The average scores for each specific element were calculated, and based on the obtained score, based on the elements with an average score of three or higher were among the significant items and were determined as the final elements. All statistical analyzes were typically performed using SPSS ver.23 software.

### Application design

The design of the mobile-based self-care application was conducted based on review of the literature in the fields of design and an analysis. Then, in consultation with the application developers, the extracted information elements and required functionalities were reviewed and finalized. Accordingly, the "Use Case" diagrams were designed and then, the application was developed. The Java programming language was used to design an application based on the Android operating system in the Android Studio programming environment.

### Application evaluation

To evaluate the usability of the application and determine the level of users' satisfaction with the application, the standard PSSUQ and NAU questionnaires were used [26, 27]. The validity and reliability of PSSUQ and NAU questionnaires have previously been confirmed in other studies with Cronbach's alpha coefficient of 0.98 and 0.60, respectively, indicating its acceptable reliability and high interpreting capability [28, 29]. The last version of PSSUQ questionnaire, version 18.3, was rewritten in 2002 in the form of sentences in a 7-point Likert scale, ranging from 0 (strongly disagree) to 7 (strongly agree). However, Nielsen’s attributes of usability are a real simple 7-points Likert scale questionnaire which is composed of five items [29].

**Fig. 1:**
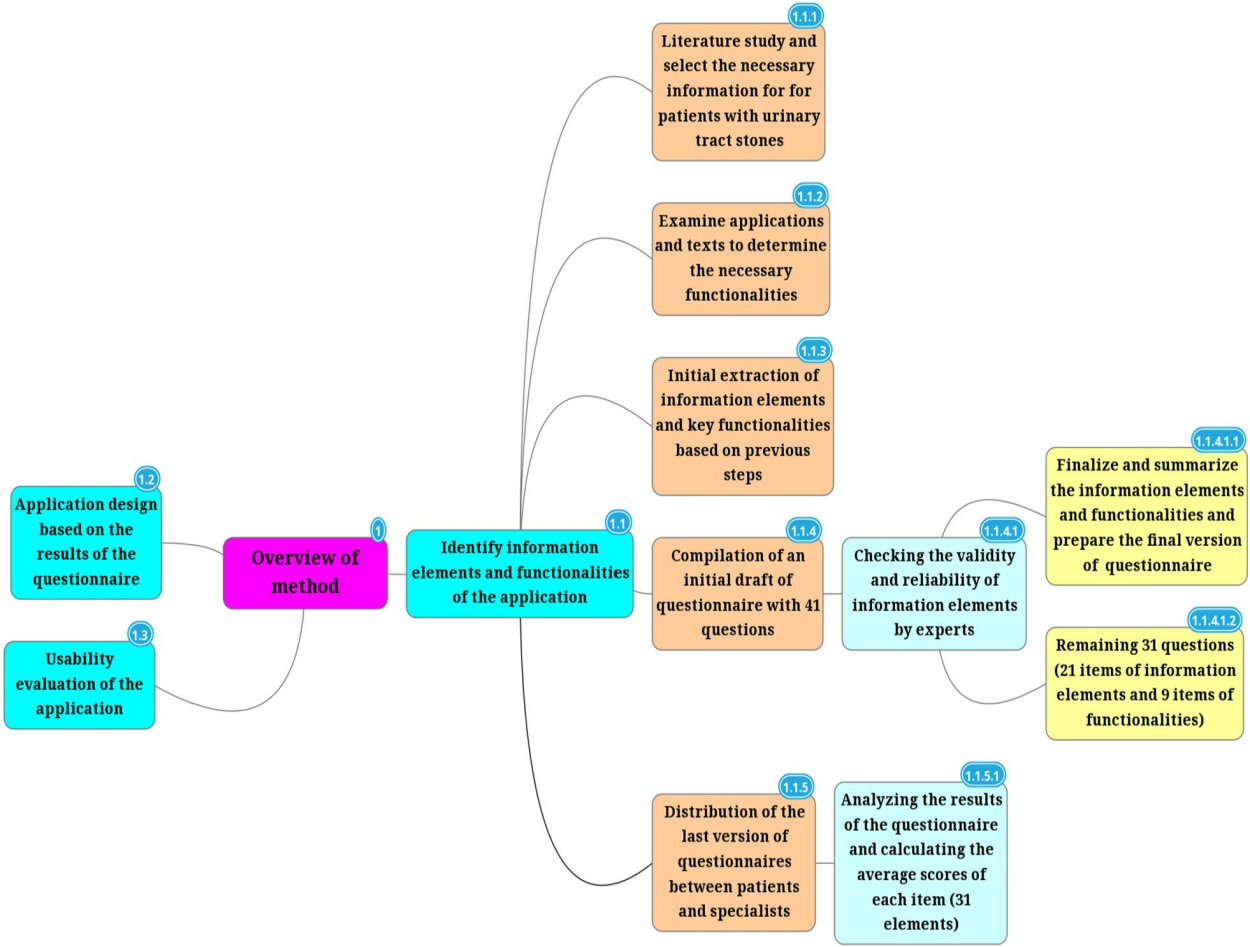
Mind map of our methodology design

Finally, these questionnaires were given to 15 physicians, nurses and medical staff caring for patients with urinary tract stones at the Shahid Hasheminejad’s Kidney and Urinary Tract Center. Since Hasheminejad Hospital is a center of nephrology and urology in Tehran, its physicians and medical staff were recruited for this survey; the sampling method was convenience sampling. The results obtained from the questionnaires were entered into SPSS-23 software for analysis using descriptive statistics. The process and phases of the conducted poll in detail are presented in Fig. 1.

### Ethical aspects

All methods were carried out in accordance with relevant guidelines and regulations. The methodology for this study was approved by the Ethics committee of Tehran University of Medical Sciences (Ethics approval number: IR.TUMS.SPH.REC.1395.1880). All participants (or their legal guardians) were provided verbal informed consent for all stages of study and the ethics committee approved this procedure.

## Results

### Demographic information of patients participating in the survey

Among the 101 participated patients with urinary tract stones in the survey, there were more men than women, so that men with 80 (79.2%) and women with 21 (20.8%) were formed the gender of individuals. The weight and height of patients with a history of urinary tract stones by year are shown in Table 1. In terms of the location of the stone, the results showed that 86 patients (85.1%) had kidney stones, six (5.9%) had ureteral stones, and nine (8.9%) had a history of stone formation in both locations. In terms of drug use, 76 patients (75.2%) do not take drugs, and 25 patients (24.8%) take drugs to prevent or treat stones or have a history of drug use. In terms of comorbidity, 81 patients (80.2%) reported not having comorbidity, and 20 patients (19.8%) had comorbidity.

**Table 1:** History of stones, the weight and height of patients

Variables	Number (persons)	Mean	SD	Minimum	Maximum
History of urinary tract stones (years)	97	6.53	7.97	0	30
Weight (kg)	101	78.7	14.54	52	110
Height (cm)	101	172.34	9.86	151	188

### Demographic information of specialists (physician, nurse, other related medical staff) participating in the survey

Table 2 shows the information about the specialists and treatment staff in the survey. This table shows the type of job and work experience by year in the field of care and history of providing services to urological patients.

**Table 2:** Descriptive information of the medical staff participating in the survey

Participants	Number (persons)	Mean of work experience (yrs.)	SD	Minimum	Maximum
Physician	32	6.53	7.97	1	22
Nurse	11	20.45	10.27	4	30
Other staff	6	8.50	4.92	4	13

### Information elements and key functionalities

As it is known, due to that the unnecessary elements and functionalities were removed in the first stage (i.e., collecting experts' opinions to confirm the validity and reliability of the elements of the initial draft of the questionnaire), all the remaining elements in this stage have average scores above three (out of four) and were considered in the design of the application program (for 150 participants: 101 patients, 32 physicians, 11 nurses, and six other specialists). All information elements and application functionalities included in the final questionnaire are listed in the table below (Table 3).

**Table 3:** The mean and standard deviation of scores for information elements and the application functionalities in the questionnaire

	Mean of scores	Standard deviation (SD)
1. Information elements		
1-1. The most common causes of urinary stones	3.65	0.55
1-2. Epidemiological risk factors in the formation of urinary stones	3.42	0.77
1-3. Pathogenesis of urinary stones	3.04	0.89
1-4. Clinical manifestations of urinary stones	3.36	0.74
1-5. Location classification of urinary system stones	3.21	0.92
1-6. Cause of the formation of calcium stones	3.42	0.78
1-7. Cause of formation of uric acid stones	3.53	0.73
1-8. Cause of formation of infectious stones	3.64	0.59
1-9. Cause of cystine formation stones	3.31	0.88
1-10. How to laboratory test a patient with recurrent stones	3.36	0.68
1-11. Important treatments for calcium stones	3.55	0.58
1-12. Important treatments for uric acid stones	3.51	0.64
1-13. Important treatments for infectious stones	3.58	0.55
1-14. Important treatment methods in cystine stones	3.45	0.71
1-15. Important differences in the treatment of urinary stones in children	3.49	0.71
1-16. Extracorporeal lithotripsy	3.66	0.55
1-17. Transurethral lithotomy (crushing from the urethra)	3.50	0.72
1-18. Percutaneous nephrolithotomy (removal of stones through the skin)	3.52	0.68
1-19. Laparoscopy in urinary stones	3.34	0.86
1-20. The role of environmental factors in the treatment of patients with urinary stones	3.53	0.67
1-21. The role of nutrition in the formation of urinary tract stones	3.74	52.0
2. Functionalities of the application		
2-1. Medicinal reminders	3.78	0.41
2-1. Fluid consumption reminder (adjusted according to specific conditions)	3.72	0.44
2-3. Schedule the next visit	3.32	0.94
2-4. Reminders of periodic visits	3.93	0.88
2-5. Reminders of laboratory tests and radiographs	3.34	0.85
2-6. Reminders of the date of surgery or hospitalization or surgical stone crushing	3.37	0.93
2-7. Medical Center Information	3.45	0.73
2-8. Information of urologists and nephrologists	3.58	0.55
2-9. Searching capability for the risk of stone formation for food items	3.70	0.51

### Designing the self-care application

**Fig. 2:**
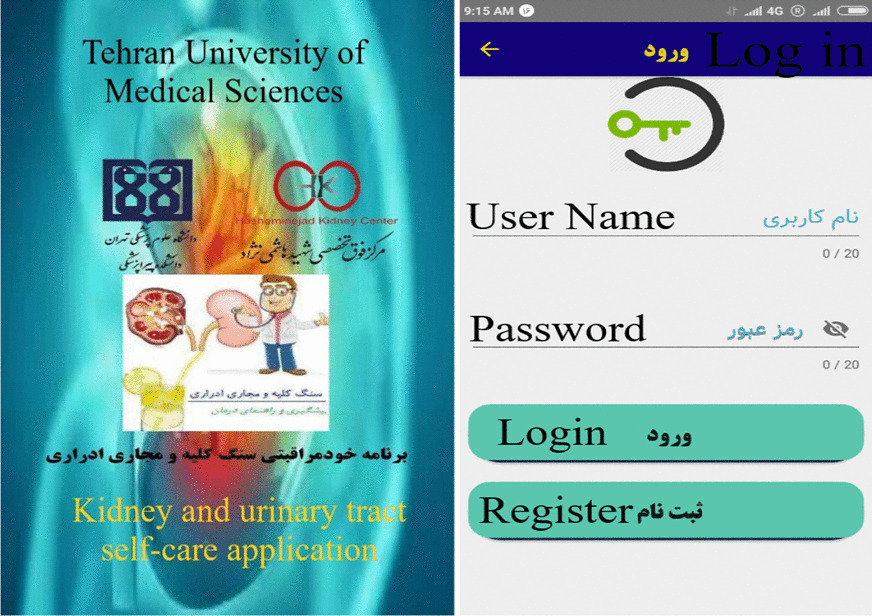
The first page of the splash application

After the need assessment of educational information and capabilities or functionalities required for designing the application, the necessary elements for managing and controlling urinary tract stones were determined and the application was designed in Android Studio software in Java language. This application has the following sections, which are presented respectively:

The first page of the application (splash page in Fig. 2) is displayed to the user for 2.5 seconds immediately after opening the application and then disappears. Figure 2 was depicted by research authors.

**Fig. 3:**
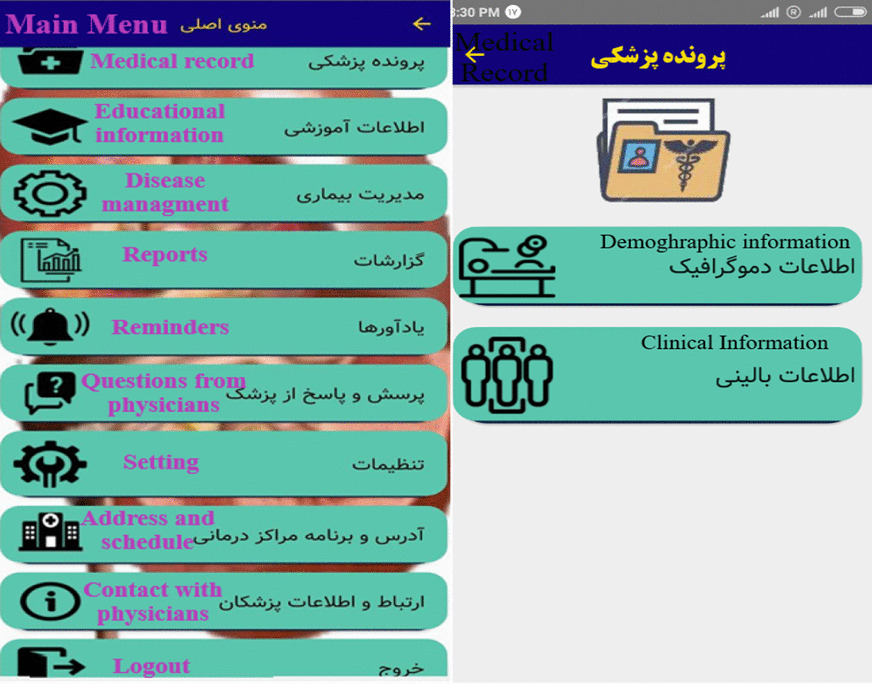
The main page of application

**Fig. 4:**
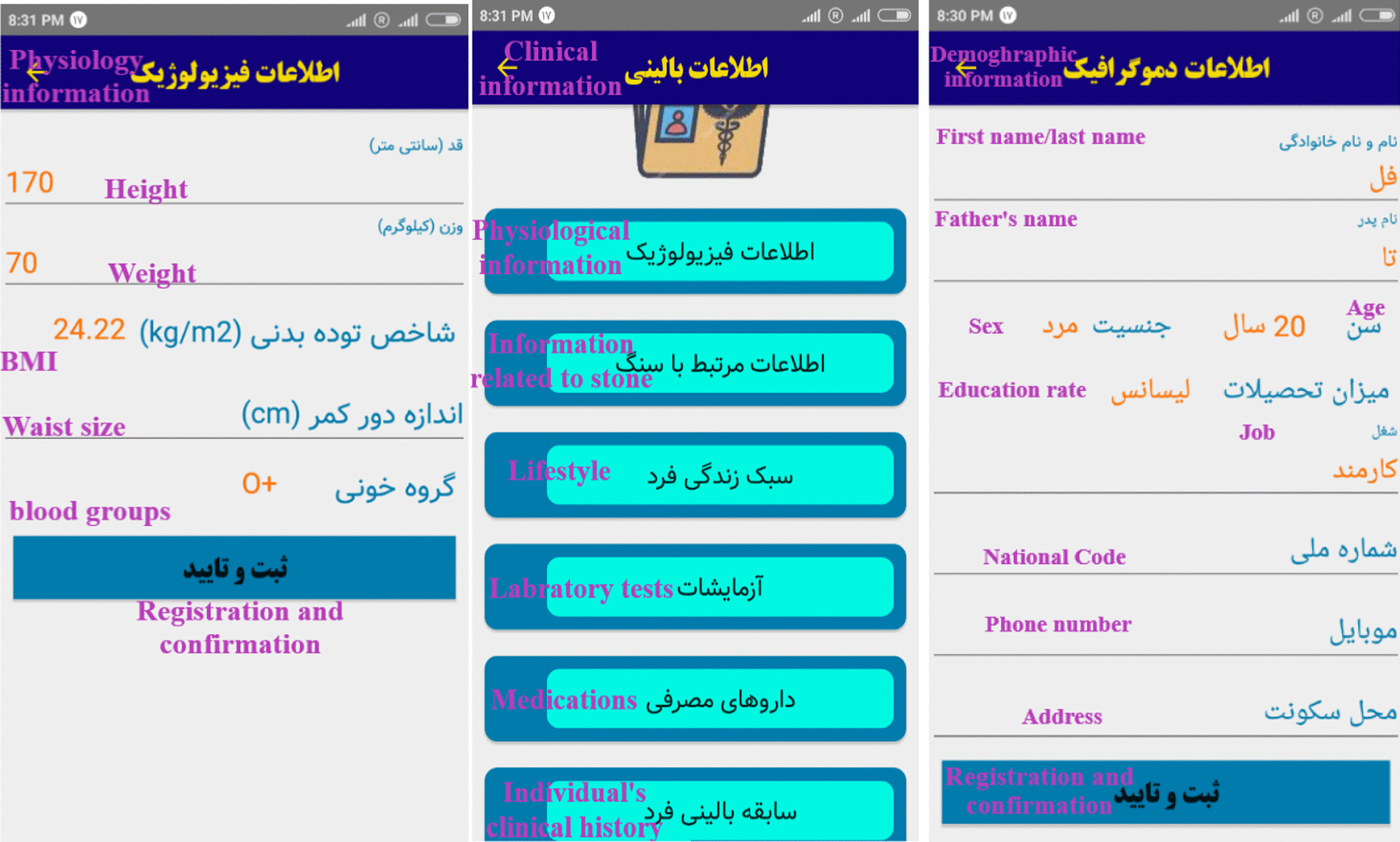
Sub-sections of medical record

First, the users must register their details in the application and created a username, and then the main page related to the application is shown as Fig. 3. The main menu of the application consists of ten sections with special features and facilities. Each section plays an important role separately based on the needs of patients identified in the survey. These sections are essential components for self-care, control and treatment of urinary tract stones. Figure 4 shows the sub-sections of medical record. Figures 3 and 4 were depicted by research authors too.

### Usability evaluation of the self-care application

**Fig. 5:**
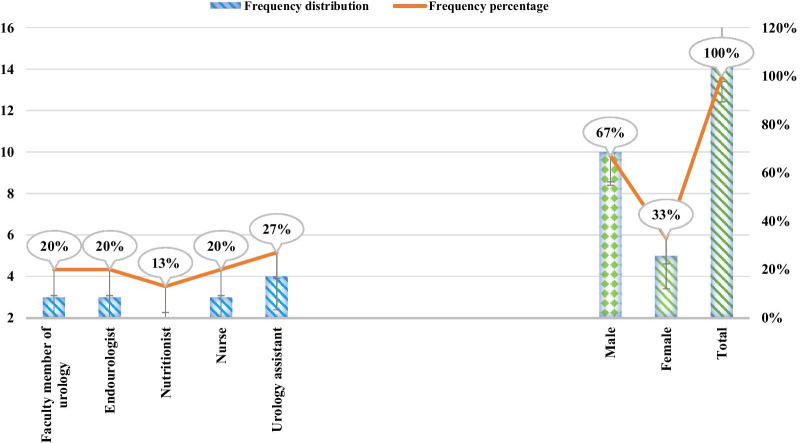
Demographic information of the experts by gender and frequency distribution of them in terms of specialty and position

A total of 15 experts participated in the study to answer the part of questionnaire that was related to evaluating the usability of smartphone-based self-care application for patients with urinary tract stones. The frequency distribution of experts who evaluated the usability of the urinary tract stones application in terms of gender is shown in Fig. 5. The frequency distribution of experts evaluating the usability of urinary tract stones application in terms of expertise and position is shown in Fig. 5 too. The frequency distribution of experts evaluating the application in terms of specialty and position is as follow; urology faculty members (n = 3), endo-urology fellowship (n = 3), urology assistant (n = 4), nurse caring for patient with urinary tract stones (n = 3) and nutritionist (n = 2).

The frequency of experts with more than twenty years of work experience was (n = 3, 19%), between ten to twenty years of work experience was (n = 4, 27%), between five to ten years of work experience was (n = 4, 27%) and less than five years of work experience was (n = 4, 27%).

Results of the application usability evaluation in Table 4 show the frequency distribution of responses of the participants in the application evaluation phase. Based on the answers and opinions of experts, the results of evaluating the usability of the application were classified into four different categories, including the level of convenience and user friendliness of the application, the quality of information provided to the user, the quality of system user interface from the user's point of view, and the overall performance of the application. The mean score obtained in this section was 6.25 from 7, consequently it can be concluded that, the experts who evaluated the application’s usability evaluated the content and overall performance of the application at a "good" level.

**Table 4:** Evaluation of the application’s usability by experts based on PSSUQ questionnaire

	Question	Questions	Mean (from 7)	Standard deviation (SD)
System usefulness	1	I am generally satisfied that I can work with this app easily.	5.8	0.86
	2	Using this app, the necessary scenarios in kidney stones can be completed more effectively.	5.7	1.03
	3	I think it is very easy to learn how to work with this app.	6.2	0.77
	4	I feel comfortable working with this app.	6.46	0.74
	5	Using this app can speed up the completion of tasks, disease management and kidney stone-related affairs.	6.15	0.84
	6	Using this app can affect the overall performance of kidney stone patients.	6.30	0.80
Overall	7	I believe that by using this application, people can be more effective in self-care of urinary tract stones.	6.27	0.88
Information quality	8	This app provides errors and messages to the user in such a way that I know exactly how to fix the problem.	6.21	0.75
	9	Whenever I have a problem while working with the app, I can easily and quickly return to the app.	6.40	0.73
	10	The information provided to the user in this app (such as guides, suggestions and errors) has made it much clearer to work with this app.	6.27	0.88
	11	In this app, I can easily obtain the information I want and get transferred to the desired section.	6.20	0.86
	12	The information provided by this app is completely understandable.	6.46	0.74
	13	App information and guides are effective in completing the various sections and scenarios of the app.	5.93	0.70
Interface quality	14	How to place and arrange information on different pages of the app is very clear and convenient.	6.60	0.63
	15	Design of the application’s display is appropriate and beautiful and I can interact well with the app.	6.00	0.75
	16	I like the look of the app and its interface.	6.46	0.74
	17	The app had the capabilities and functions that I expected.	6.66	0.48
Overall	18	I am generally satisfied with this app.	6.60	0.63
	Total		6.25	0.75

According to the results presented in Tables 5, the evaluation of application’s usability showed that the experts evaluated the application in four aspects separately. The highest score of 6.43 from 7 was related to the quality of system user interface from the user's point of view and the overall performance of the application, and then the score of 6.10 from 7 was related to two aspects of the degree of convenience and user friendliness of the application from the experts’ point of view. They also evaluated all aspects of the application at the "good" level.

**Table 5:** The mean score of applications’ usability from the experts’ point of view based on the different parts of the questionnaire

No	Classification of evaluation dimensions	Mean score (from 7)	Standard deviation
1	The degree of convenience and user-friendliness of the app	6.10	0.84
2	The quality of information provided to the user	6.24	0.77
3	The quality of system user interface from the user's point of view	6.43	0.65
4	Overall performance of the app	6.43	0.75
	Total	6.25	0.75

Also, using NAU questionnaire, the results of application usability evaluation are given in Table 6. The mean score of the NAU questionnaire showed that the application was also acceptable in terms of exploratory evaluation. The highest score with this evaluation method was related to "personal satisfaction rate" with the mean score of 6.61 and the lowest score was related to "learning ability" and "ability of error display and accuracy rate" with the mean score of 5.66. However, for the actual evaluation of the application’s usability, the usage time must be longer in order to obtain an ideal view of the application evaluation.

**Table 6:** The mean score of application’ usability from the experts’ point of view based on NAU questionnaire

No	Classification of evaluation dimensions	Mean score (from 7)	Standard deviation
1	Learning ability	5.66	0.72
2	Efficacy	6.33	0.81
3	Memorability	6.25	0.73
4	Ability to display errors and accuracy rate	5.66	0.99
5	Subjective satisfaction	6.61	0.63
	Total	6.10	0.78

## Discussion

In general, the evaluation of application’s usability by experts showed that the usability of the application for the use of patients with urinary tract stones is at an acceptable level.

Valente et al. [30] in their study showed that reporting the level of water consumption is one of the most important factors in preventing the formation of urinary tract stones. The ability to record water consumption in smartphone-based applications helps urologists to receive appropriate information and prescribe correct medications, which improve the quality of life of the patient [31]. The designed application also has the capability to record the water consumption and urine pH, which can be used to better view the patient's condition by using graphic reports. Stevens et al. [32] reviewed the smartphone applications for patients with urinary tract stones on 42 applications and showed that 79% of the 42 applications were designed for patient use, and among them 22 (52%) applications were related to patient awareness and information, 9 applications (21%) were related to occupational health resources, 4 applications (10.4%) were related to patient diet recording, 4 applications (10.4%) were related to patient counseling about herbal medicines, 2 applications (5%) were related to patient counseling about uric acid and gout, and 1 application (2%) was related to medical advertisements. Also, the review of this study showed that 15 applications (36%) of the 42 applications clearly had the contents that were prepared by health care professionals. Therefore, in terms of cost, 21 applications (50%) out of 42 applications were free of charge [32]. In designing the present study, we tried to maximize the participation and cooperation of the specialists in the field of urinary tract stones and the researcher in the application development, and also the use of experiences and opinions of physicians, nurses and other related specialists. By using patients' clinical records and surveying the patients themselves, we also tried to gain an understanding of patients' needs in order to develop a useful and comprehensive application for users. This factor distinguishes the present study from Stevens' research.

In a study entitled: “Designing and creating an Android-based psoriasis self-care application”, Masourian et al., used the Java programming language and the SQLite database in the Eclipse software environment [33]. The programming language and database used to store data in this study are similar to the present study, but the software used to design the application in the present study is different from the software used in the Masourian et al study. In another study entitled: “Design and evaluation of a mobile application to assist the self-monitoring of the chronic kidney disease in developing countries”, Sabrinho et al., used the Java programming language in the Android Studio programming environment and also the SQLite database to store information [34], which is consistent with the present study and the evaluations of this mobile-based app were approved by the Brazilian ethics committee of the UFAL and carried out between 2015 and 2016.

Agapito et al., conducted a study entitled: “DIETOS: A dietary recommender system for chronic diseases monitoring and management”, used Cooperative User Modeling to design software for mobile phones with Android operating system. In particular in this web-based system, the graphical interface is written in HTML5, CSS, and JQuery, whereas the server sides of DIETOS data querying and presentation are written by using the PHP (5.5.31 version) [35]. Hsiu-Wen Lin et al. developed a comprehensive personal health diary app for patients with chronic kidney disease. This study was developed in three main steps: analyzing information requirements by running several surveys, designing a system prototype, and evaluating this app. The results of this investigated study are consistent with our research results in terms of extracting the requirements and capabilities or functionalities of the system [36].

Alexander et al. (2019) stated that despite various applications in this field and with the growth and development of information technologies in the not too distant future, better systems and applications will be provided. Social media and telemedicine provide patients and urologists with accessible information, and bring them closer together for better treatment and management of urinary tract stones. The rate of non-adherence to treatment in kidney stone patients is similar to other chronic patients. Many of these patients suffer from symptoms of lower urinary tract disease, recurrent infections, and chronic pain. Hence, software applications increase adherence to treatment, prevention and recurrence of kidney stones, as well as possibility of telemedicine [14]. The usability of the application in changing the pattern of fluid consumption was also examined [37], indicating that out of 50 applications that could be installed and used in smartphones, only in 40% of the applications, all factors related to changing the fluid consumption behavior were considered, which indicates that not every application on the open market provides the minimums.

The strength of our study is the survey of experts and patients in the first stage to identify the information elements and functionalities of the application, which led to the generalization of our work, and the main framework of the application was developed based on the patients' needs; hence, our application is a patient-centered mHealth application. In the absence of user feedback from patients in the evaluation phase, their insights were shared that they provided in the initial survey and those features are captured within the application. It is noteworthy that surveys of patients and specialists were conducted in one of the country's most advanced urological and nephrology centers. Nevertheless, in this study, we faced the following limitations; one of the most significant limitations of our study is that in the self-care application usability evaluation phase, we were unable to survey patients due to time and budget problems. The usability evaluation of the designed application by patients can be suggested for future studies. Another shortcoming of this study is that only 15 specialists from one hospital evaluated the self-care application. This study was performed at Tehran University of Medical Sciences and was evaluated in Hasheminejad Hospital. These clinical and academic environments support the customer-provider relationship. It should be noted that there is no repository of vetted health apps at the national level in Iran, though the current application is academically designed, and this can guarantee the patient's rights.

## Conclusion

The results showed that, the smartphone-based self-care application for patients with urinary tract stones was designed based on patient needs assessment and the participation of clinical and health information specialists, which is available to users as an Android-based application. This self-care application includes the following sections; recording demographic information and clinical characteristics of patients, educational information on urinary tract stones, disease management, recording and reporting of water consumption, urine pH, test results and radiology photos, the possibility of attaching clinical documents, dietary recommendations based on the individual characteristics of the user, reminders (reminders of drug use, water consumption, periodic visits, para-clinical procedures, and stent removal date to prevent infection), introducing physicians and medical centers for counseling and medical services, the possibility of communicating with the physician, and a set of features provided to the user in an integrated way to control and prevent the recurrence of stones.

This application can be considered as a model for designing and creating better systems and applications to extend the patient self-care, education and management of chronic diseases and facilitate active participation of the patient in promoting health and improving the public lifestyle.

Despite the facilities and features provided by this application for patients with urinary tract stones, it is still possible to provide new functionalities and update the application with research to make it more efficient for these patients.

## Data Availability

All data generated or analyzed during this study are included in this published article**.**
